# *Gardnerella vaginalis* as a Cause of Bacterial Vaginosis: Appraisal of the Evidence From *in vivo* Models

**DOI:** 10.3389/fcimb.2020.00168

**Published:** 2020-04-24

**Authors:** Sydney Morrill, Nicole M. Gilbert, Amanda L. Lewis

**Affiliations:** ^1^Department of Molecular Microbiology, Washington University School of Medicine, St. Louis, MO, United States; ^2^Center for Women's Infectious Disease Research, Washington University School of Medicine, St. Louis, MO, United States; ^3^Department of Obstetrics and Gynecology, Washington University School of Medicine, St. Louis, MO, United States; ^4^Center for Reproductive Health Sciences, Washington University School of Medicine, St. Louis, MO, United States

**Keywords:** *Gardnerella vaginalis*, animal model, bacterial vaginosis, dysbiosis, co-infection

## Abstract

Koch's postulates dictate the use of experimental models to illustrate features of human disease and provide evidence for a singular organism as the cause. The underlying cause(s) of bacterial vaginosis (BV) has been debated in the literature for over half a century. In 1955, it was first reported that a bacterium now known as *Gardnerella vaginalis* may be the cause of a condition (BV) resulting in higher vaginal pH, thin discharge, a fishy odor, and the presence of epithelial cells covered in bacteria. Here we review contemporary and historical studies on BV with a focus on reports of experimental infections in human or animal models using *Gardnerella vaginalis*. We evaluate experimental evidence for the hypothesis that *G. vaginalis* is sufficient to trigger clinical features of BV or relevant health complications associated with the condition. Additionally, we evaluate *in vivo* models of co-infection employing *G. vaginalis* together with other bacterial species to investigate evidence for the hypothesis that *G. vaginalis* may encourage colonization or virulence of other potential pathogens. Together, these studies paint a complex picture in which *G. vaginalis* has both direct and indirect roles in the features, health complications, and co-infections associated with BV. We briefly review the current taxonomic landscape and genetic diversity pertinent to *Gardnerella* and note the limitations of sequence-based studies using different marker genes and priming sites. Although much more study is needed to refine our understanding of how BV develops and persists within the human host, applications of the experimental aspects of Koch's postulates have provided an important glimpse into some of the causal relationships that may govern this condition *in vivo*.

## Introduction

Bacterial vaginosis (BV) is a dysbiosis—a condition of the vaginal microbiome that has been associated with a wide variety of adverse health outcomes. The condition is characterized by low levels of “healthy” lactobacilli and overgrowth of diverse bacteria from other taxonomic groups, including *Gardnerella, Atopobium, Mobiluncus, Prevotella, Bacteroides, Anaerococcus, Peptostreptococcus, Sneathia, Leptotrichia*, and members of the class *Clostridia*, among others (Ravel et al., 2011; Srinivasan et al., 2012). BV has been associated with higher risk of sexually transmitted infections (Wiesenfeld et al., 2003; Brotman et al., 2010; Van De Wijgert, 2017), urinary tract infections (Harmanli et al., 2000; Hillebrand et al., 2002), post-surgical complications (Watts et al., 1990), infertility (Spandorfer et al., 2001), pregnancy losses (Ralph et al., 1999), preterm birth (Svare et al., 2006), intrauterine (Di Paola et al., 2017; Ádám et al., 2018) and intraamniotic infections (Silver et al., 1989), as well as cervical infections, dysplasia, and cancer (Di Paola et al., 2017; Laniewski et al., 2018; Brusselaers et al., 2019). These conditions and health complications associated with BV and its member bacteria can be caused not only by bacteria, but also by eukaryotic (e.g., trichomoniasis) (Jarrett et al., 2019) and viral (e.g., HIV, HSV, HPV) pathogens. In addition, women with diverse BV-like microbiomes are more likely to exhibit signs of genital inflammation (Lennard et al., 2017) and vaginal colonization by other potential pathogens such as beta-hemolytic streptococci (Cherpes et al., 2005) and *Fusobacterium nucleatum* (Hillier et al., 1993; Hill, 1998; Hitti et al., 2001). BV is characterized by reduced numbers of lactobacilli and overgrowth of a polymicrobial consortium often containing large numbers of *Gardnerella vaginalis* (Shipitsyna et al., 2013; Balashov et al., 2014). Despite all of this, many/most women do not report symptoms of BV to their providers, even in settings where the clinical signs and/or inflammatory markers are evident (Masson et al., 2019).

First reported as “*Haemophilus vaginalis,”* the organism now known as *Gardnerella vaginalis* was reported to be the sole cause of clinical signs and symptoms now used to diagnose BV (then referred to as non-specific vaginitis, NSV) (Gardner and Dukes, 1954, 1955). However, controversies surrounding the clinical significance of *Gardnerella* have abounded since its debut in the literature in the 1950s. Today the pendulum has swung in the opposite direction. While some believe that *G. vaginalis* may be a sole causal contributor to BV (Schwebke et al., 2014b), others have been skeptical of this and consider *G. vaginalis* to be one of many within the BV consortium (Hickey and Forney, 2014; Schwebke et al., 2014a). Recently, a more complex model has been proposed, taking into account recent efforts to build animal models for BV and arguing that relationships between multiple microbes, including *G. vaginalis*, may underpin the condition (Muzny et al., 2019a).

Here we review and consider the implications of contemporary and historical findings from studies reporting experimental infections *in vivo* using *Gardnerella vaginalis*. From the early experimental studies in women, to more recent attempts to replicate clinical signs and associated health complications of BV in small animal models, the existing studies paint a complex picture that reflects the biology of the mammalian vagina and the organisms that colonize it. In this review, we will evaluate the evidence from experimental inoculations in humans and other animals that *G. vaginalis* (on its own, or together with other organisms) has a causal role in generating features or complications that have been linked with BV. We also review and discuss the interpretations of some of the earlier experiments in light of our current appreciation about *Gardnerella* and BV, specifically considering the genetic diversity among *Gardnerella* strains (Ahmed et al., 2012; Janulaitiene et al., 2018; Vaneechoutte et al., 2019).

## Historical Context and Current Controversies

Originally the diagnostic term “non-specific vaginitis” (NSV) was used to describe vaginal symptoms of unknown etiology, but Gardner and Dukes suggested in the 1950's that >90% of NSV cases were caused by a single organism-*G. vaginalis* (Catlin, 1992). A substantial body of clinical literature links both NSV (and as it later became known BV) to adverse health outcomes. Although the term “vaginitis” suggests otherwise, NSV was not typically associated with canonical signs of inflammation such as redness or swelling. Even in the 1950s, Gardner and Dukes realized that “argument may be advanced against calling this infection an inflammatory disease as the term vaginitis implies; however, it must continue to be included in this category until such time as a more suitable term has been proposed and accepted” (Gardner and Dukes, 1959). It would be decades later before “bacterial vaginosis” (BV) started to be used in the literature as a diagnostic term that more accurately represented the condition in question. Around the time “BV” started to be used, Amsel et al. outlined a set of clinical criteria, now known as the Amsel criteria, to diagnose NSV (Amsel et al., 1983). Today, the Amsel criteria is still used clinically for the diagnosis of BV (C.f.D.C.a. Prevention, 2015). Three of four of the following (Amsel) criteria are generally considered to support the diagnosis of BV: (1) a thin, homogenous discharge, (2) higher than normal pH (>4.5), (3) the presence of “clue” cells in wet mount (epithelial cells that appeared to be coated in bacteria), and (4) the detection of a fishy odor, with or without treatment of the sample with 10% potassium hydroxide. BV can also be diagnosed via the Nugent scoring method. Although high interobserver variability of the Nugent score has been noted in some studies (Forsum et al., 2008), it remains the current gold standard for laboratory-based BV diagnosis used in clinical research. First published by Nugent et al. (1991), this method does not rely on clinical signs of BV, but instead uses Gram-stained smears of vaginal fluid to evaluate the balance of bacterial morphotypes present in the genital tract, resulting in a score from 0 to 10 (Nugent et al., 1991). A Nugent score of 0–3 indicates a normal *Lactobacillus*-dominant microbiota, 4–6 indicates an “intermediate microbiota,” and a score of 7–10 indicates BV (Nugent et al., 1991; Amegashie et al., 2017).

Additional characteristics of BV include lower viscosity vaginal fluids, a shift in fermentation products and other metabolites, presence of the diamines putrescine and cadaverine, detectable levels of the enzyme sialidase and the liberation and overall depletion of mucosal sialic acids (Briselden et al., 1992; Catlin, 1992; McGregor et al., 1994; Olmsted et al., 2003; Lewis et al., 2013; Chappell et al., 2014; Srinivasan et al., 2015; Nelson et al., 2018). BV is still clinically considered a non-inflammatory condition, as it is not typically associated with edema of vaginal tissue (Mitchell and Marrazzo, 2014) nor increased numbers of neutrophils in the cervicovaginal space (Giraldo et al., 2012). However, several studies have reported increased levels of pro-inflammatory cytokines such as IL-1β, IL-6, IL-8, and IP-10 (among others) in vaginal specimens from women with BV as compared to “healthy” controls (Cauci et al., 2002, 2008; Hemalatha et al., 2012). The apparent discrepancy between clinical findings (lack of neutrophils/overt inflammation) is not fully understood (Forsum et al., 2005). High resolution studies evaluating relationships between chemokine/cytokines and specific types of communities or member bacterial taxa associated with BV have found that larger proportions of *Prevotella* and other microbes were associated with a more pro-inflammatory phenotype and increased risk of adverse health outcomes (Anahtar et al., 2015; Gosmann et al., 2017; Lennard et al., 2017; Shannon et al., 2017; Fettweis et al., 2019). However, in general, high levels of neutrophils present in vaginal secretions is indicative of other conditions such as “desquamative inflammatory vaginitis” or “aerobic vaginitis” (Donders et al., 2002; Mason and Winter, 2017). Finally, we emphasize that not all cases BV are characterized by the same signs/symptoms or the presence of clue cells and no single species is a universal marker of BV (Srinivasan et al., 2012).

*Gardnerella vaginalis* has had a long history of controversy related to its taxonomy and clinical significance, some of which continues to this day. Around the same time the organism was isolated by Gardner and Dukes from the vaginas of women with nonspecific vaginitis (NSV) (Gardner and Dukes, 1954, 1955), it was also identified as a “*Haemophilus-*like” bacterium found in patients with cervicitis and prostatitis (Leopold, 1953). These first reports classified it as a *Haemophilus* species because they found it negative by Gram staining and unable to grow on agar without blood (Turovskiy et al., 2011). However, later studies revealed that *H. vaginalis* did not require hemin or nicotinamide adenine dinucleotide (NAD) for growth, making it clear that the organism was not actually a member of the *Haemophilus* genus. Additionally, the bacterium appeared to sometimes have a positive Gram staining reaction. For these reasons *H. vaginalis* was reassigned to the *Corynebacterium* genus, and renamed *Corynebacterium vaginale* (Catlin, 1992). This name too proved to be inaccurate, as unlike *Corynebacterium* species, the bacterium was catalase negative and did not have arabinose in its cell wall (Turovskiy et al., 2011). In 1980, two large taxonomic studies used DNA-hybridization, electron microscopy, and a variety of biochemical methods to show that the bacterium lacked close similarity with any previously established genera (Greenwood and Pickett, 1980; Piot et al., 1980; Catlin, 1992). This lead to the proposal of a new genus, *Gardnerella*, and the name *Gardnerella vaginalis* for the bacterium (Greenwood and Pickett, 1980).

Despite early confusion about the taxonomy of what came to be known as *Gardnerella*, Gardner and Dukes believed they had found the sole etiological agent of most cases of NSV. The 1955 paper reported that among the (Caucasian) women from Gardner and Dukes gynecology practices, *H. vaginalis* was isolated from the lower genital tract of 127 out of 138 women diagnosed with NSV (92%), but none of the 78 healthy women examined. Gardner and Dukes argued that the vast majority of NSV cases were really cases of *H. vaginalis* infection (Gardner and Dukes, 1955). But, not long after *H. vaginalis* was discovered as a potential causative agent of NSV, it became a point of controversy. This topic remains controversial to this day, with some recent studies using sensitive molecular techniques suggesting that most women (including >60% without BV) have *Gardnerella* colonizing the vagina, although women without BV had ~4 orders of magnitude lower levels compared to women with BV (Balashov et al., 2014; Cox et al., 2015). Of note, primer sets that target variable regions 1 and 2 of the gene encoding 16S often see lower relative levels of *Gardnerella* in women with BV when compared to studies that target variable regions 3 and/or 4 or that use culture-based studies (Graspeuntner et al., 2018). It has been recently emphasized that 16S gene sequences cannot differentiate the genetically divergent subsets of *Gardnerella* and that other methods (e.g., cpn60 sequencing) are more effective in this regard (Hill et al., 2019). Regardless of the method used to detect *Gardnerella* in women, whether the organism is a causal factor in BV cannot be determined by observational study. A number of experimental studies in humans and other animals, which we review here, have been conducted in an attempt to address this question.

*Methodology in brief:* The purpose of this review is to evaluate the experimental evidence for *G. vaginalis* as a causal factor in generating the features or health complications that have been associated with BV. Thus, in general, we sought to focus on publications that had a similar experimental intent. While this was not intended to be a systematic review, we first searched PubMed using the search string: [(“*Gardnerella*” or “*Haemophilus vaginalis*”) AND (“murine” OR “mouse” OR “mice” OR “*in vivo*” OR “animal” OR “model”)]. Some papers, especially older literature, could not be found using these specific search terms. To find additional papers relevant to the topic, we also carefully reviewed several published reviews on BV and/or *Gardnerella* that addressed animal models (Catlin, 1992; Turovskiy et al., 2011; Herbst-Kralovetz et al., 2016). Once the papers were located, they were screened for inclusion in this review based on the following criteria: (1) The papers were available in English, (2) They described an *in vivo* inoculation of *G. vaginalis* into the urogenital tract of an animal (including humans), and (3) After inoculation, experimental hosts were assessed for one or more features or health complications previously associated with BV. Based on these criteria, we excluded papers in which *G. vaginalis* was isolated from animals but never experimentally inoculated, inoculation of *G. vaginalis* was not into the urinary or reproductive tract, or the sole intent was to introduce an intervention to eliminate *G. vaginalis* colonization rather than to understand the causal or mechanistic role of *G. vaginalis* interaction with the experimental host.

## Experimental Studies *in vivo* Using *Gardnerella* Alone

### Vaginal Inoculation in Women

The earliest *in vivo* experiments using *G. vaginalis* were performed in women with the intent of demonstrating “proof of pathogenicity.” Gardner and Dukes suggested that *G. vaginalis* could trigger “bacterial vaginitis” in otherwise healthy women. Gardner and Dukes asserted that *G. vaginalis* was the sole causative agent in most cases of the condition (Gardner and Dukes, 1955). In their experimental infections, they vaginally inoculated 13 healthy female volunteers with “pure cultures” of several different strains of *G. vaginalis* that had been isolated from women with BV (although specific strains or culture conditions were not reported, see Table 1). Of the inoculated women, 10 of 13 tested negative for *G. vaginalis* after inoculation. Two women had positive cultures for *G. vaginalis* for 2–3 months, but showed none of the clinical signs of NSV previously described by Gardner and Dukes (vaginal pH >5, the presence of clue cells, thin homogenous discharge, and a decrease in vaginal lactobacilli). Only one of the 13 women was both culture-positive for *G. vaginalis* and displayed clinical signs (Gardner and Dukes, 1955).

**Table 1:** Experimental studies of *G. vaginalis* infection in humans and other primates.

**References**	**Host species**	**Bacterial strain(s)**	**Culture media**	**Culture condition**	**Culture** **time (h)**	**Dose**	**Route**	**N**	**n colonized by *G.v*.**	**Positive for features/complications of BV**
Gardner and Dukes, 1955	Human (pregnant and non-pregnant)	NR	NR	Limited oxygen	NR	NR	Vaginal	13	3/13	1/13 positive for *G.v*. & clinical signs: homogenous, odorous discharge; pH>5.0
		“Material from the vagina”	N/A	N/A	N/A	N/A	Vaginal	15	11/15	11/15 positive for *G.v*. & clinical signs: homogenous, odorous discharge; pH>5.0
Criswell et al., 1969	Human (pregnant)	*G.v*. 3299, 3309, 3310	Biphasic. Bacto Casman's Agar + 5% rabbit serum	10% CO_2_	24	2 × 10^10^	“Poured [broth cultures] into the vagina”	15	2/15	2/15 *G.v*. positive & clinical signs: gray, thin, homogenous, odorous discharge; pH >5.0, clue cells, Gram neg rods in smears
		*G.v*. 594 (ATCC 14018)			24			5	0/5	0/5 *G.v*. positive; no clinical signs (as above)
					12			9	5/9	5/9 *G.v*. positive & clinical signs (as above)
Johnson et al., 1984	Pig-tailed macaque	*G.v*. 584, 614	Peptone-starch-dextrose broth + 10% horse serum	NR	24	5 × 10^6^- 1 × 10^7^	Intravaginal (catheter or pipette)	10	10/10	0/10 had clue cells; increased pH; non-volatile fatty acids
	Tamarin	*G.v*. 584				3 × 10^6^		4	0/4	0/4 had increased pH
	Chimpanzee	*G.v*. 812 and 958	Bordet-Gengou agar	NR	48	5 × 10^7^- 1 × 10^8^		3	0/3	0/3 had clue cells; increased pH
Mårdh and Møller, 1984	Grivet monkeys	*G.v*. L824 LCR L100 (long curved rod) SCR L1599 (short curved rod)	NR	Anaerobic or 10% CO2	48	2 × 10^9^	Intravaginal (swab)	8	2/8	Profuse, thin gray discharge observed in 2/2 animals infected with *G.v*. + LCR, 0/3 mono-infected animals. *G.v*. recovered only from *G.v*. + LCR infected animals. No clue cells, increased odor, or elevated vaginal pH observed in any animals

A second experiment was performed with 15 healthy female volunteers, but the women were inoculated directly with vaginal material from patients with NSV rather than cultured *G. vaginalis*. This inoculation resulted in NSV signs in 11 of 15 women. In all of the patients that displayed clinical symptoms, *G. vaginalis* was recovered by culture. Gardner and Dukes attributed the low-incidence of vaginitis upon infection with pure *G. vaginalis* cultures compared to direct inoculation with vaginal material, to a loss of viability during serial passaging of the bacteria (i.e., repeated culturing of the strain in the laboratory) (Gardner and Dukes, 1955). However, looking back from a modern perspective, these findings have other potential explanations. For example, culture conditions are not stated and viability of the inoculum used to infect women was not evaluated; limited viability could have been due to growth phase rather than serial passage. Alternatively, perhaps this was an early indication that other organisms in addition to *G. vaginalis* may play a role in BV. Consistent with the reproducible initiation of BV by exposure to vaginal material (Gardner and Dukes, 1955), a number of modern investigations have shown that specific sexual practices that would result in a similar exposure to vaginal material are linked with higher risks of BV, including the sharing of sex toys between women who have sex with women (Bradshaw et al., 2014; Olson et al., 2018; Muzny et al., 2019b).

Criswell et al. (1969) reported another attempt to induce NSV in women via inoculation with pure cultures of *G. vaginalis*. Twenty-nine pregnant volunteers were vaginally inoculated with one of four different *G. vaginalis* strains (about 2 × 10^7^ CFU), which had been grown for either 12 or 24 h in liquid media. The women were observed for development of NSV (referred to as “*H. vaginalis* vaginitis”). Only two of the 20 subjects inoculated with a 24-h culture of *G. vaginalis* developed symptoms, but five of the nine women that were inoculated with a 12 h culture showed clinical signs of *H. vaginalis* (see Table 1). The primary conclusion by Criswell et al. was that these results confirmed the earlier human experiments and hypotheses put forward by Gardner and Dukes, namely that *G. vaginalis* infection alone could trigger NSV. Their findings suggested that the age of the inoculum affected the ability of *G. vaginalis* to colonize the host resulting in clinical signs of NSV (Criswell et al., 1969). We will explore this concept further in Section Discussion.

### Vaginal Inoculation in Non-human Primates

In 1984, Johnson et al. published an attempt to model NSV in non-human primates (Johnson et al., 1984) (see Table 1). Twelve pig-tailed macaques, six tamarins, and four chimpanzees were vaginally inoculated with one of four different *G. vaginalis* strains. These strains had been collected from women diagnosed with NSV at a London clinic. The authors were unable to recover *G. vaginalis* from any tamarins or chimpanzees 5–7 days after inoculation. Only the pig-tailed macaques appeared susceptible to colonization with the strain used in this setting, as all of the inoculated animals remained culture-positive for *G. vaginalis* for 11–39 days. It was noted that the *G. vaginalis* strain used for inoculation of chimpanzees was different than the one used in macaques, and the former isolate had been extensively passaged in the laboratory, whereas the latter had only been passaged once. One of the two control macaques receiving PBS (vehicle alone) had detectable *G. vaginalis* later in the experiment, which the authors attributed to cross-contamination from a colonized animal during vaginal washing (Johnson et al., 1984).

The authors examined the infected pig-tailed macaques for clinical signs of NSV. No clue cells were observed in the vaginal smears, regardless of whether the animal was successfully colonized with *G. vaginalis*. Women with NSV had been previously shown to exhibit higher vaginal pH and a higher ratio of succinate to lactate compared to women without NSV (Amsel et al., 1983). However, the authors also did *not* see an increase in vaginal pH or higher succinate to lactate ratio in the colonized animals. This may be explained by differences in vaginal physiology and the endogenous microbes between humans and macaques. *Lactobacillus*-dominant vaginal microbiomes in women produce lactate, generating the characteristically low vaginal pH (3.8–4.5) seen in most women (Boskey et al., 1999, 2001). The authors noted that compared to these “normal” women, macaques at baseline already seemed to possess NSV-like characteristics, including a high pH (6.0–7.0) and a high succinate to lactate ratio (Johnson et al., 1984). The authors also noted a high baseline vaginal pH in the chimpanzees (5.5–6.0), and tamarins (7.0). These data suggest that non-human primates have a fundamentally different composition of microbes with different metabolic properties compared to women. More recent studies using molecular approaches have confirmed that macaque and other non-human primate vaginal microbiomes typically reflect a scarcity of lactobacilli accompanied by diverse microbial taxa, some of which are also found in human BV (Uchihashi et al., 2015; Zhu et al., 2015; Obiero et al., 2016; Miller et al., 2017). A 2010 study identified *G. vaginalis* via 16S sequencing in the vaginal microbiome of Rhesus macaques. However, *G. vaginalis* was found in only 2 of the 11 macaques and was present at relatively low levels—unlike the high abundance and prevalence of *G. vaginalis* in human BV (Spear et al., 2010).

### DNA as a Component of *G. vaginalis* Biofilms

BV can be treated with antibiotics (most often metronidazole and clindamycin) and antiseptics (e.g., dequalinium chloride) (Mendling et al., 2016). However, though initially effective in ~80% of cases, the rates of recurrence following antibiotic treatment are extremely high; >50% of women will have recurrent episode(s) within 6–12 months (Bradshaw and Brotman, 2015). One proposed explanation for these high rates of recurrence is the presence of *G. vaginalis* biofilms. Dense biofilms containing *G. vaginalis* have been identified on the vaginal epithelium of women with BV (Swidsinski et al., 2005), and these biofilms can sometimes re-form after oral metronidazole treatment (Swidsinski et al., 2008). In 2013, Hymes et al. demonstrated *in vitro* that *G. vaginalis* biofilms formed on polystyrene plates contain extracellular DNA. The biofilms could be disrupted by DNase application, resulting in a ~5-fold reduction of bacterial titers measured in biofilms. Furthermore, using a vaginal *G. vaginalis* infection model in C57BL/6 mice, the authors also demonstrated that *in vivo* DNAse treatment resulted in a >10-fold reduction in *G. vaginalis* titers in the mouse vagina after 48 h (see Table 2 for comparison to other rodent colonization models). However, the authors did not evaluate whether *G. vaginalis* forms biofilms in the mouse vagina, and colonization density was low at 48 h, even in untreated mice (Hymes et al., 2013). Other than the presence of *G. vaginalis* in the vagina, this study did not examine or report the presence of other features of BV.

**Table 2:** Experimental studies of *G. vaginalis* infection in rodent models.

**References**	**Host species**	**Bacterial Strain(s)**	**Culture Media**	**Culture Condition**	**Culture time (h)**	**Dose**	**Route**	**N**	**n colonized** **by *G.v*.**	**Positive for features/** **complications of BV**
Field et al., 1993	New Zealand & Calif. White Rabbits (pregnant)	*G.v*. ATCC 14018	V-selective agar	Increased CO2	48–72	2 × 10^4^- 2 × 10^6^	Uterine (transvag/ cervical cannula)	17	N/A	17/17 *G.v*. deciduitis; 15/17 *G.v*. intraamniotic infection; 10/17 severe neuronal injury (0 in controls); 2/17 had preterm labor; infected group had lower weight & high fetal mortality
McDuffie et al., 2002	New Zealand White Rabbits (pregnant)	*G.v*. NR	NR	NR	NR	10^7^		16	11/16	*G.v* intraamniotic (9/16), uterine (11/16), blood (7/16), fetal brain (10/16), fetal heart (6/16), and fetal lung (8/16) infection.
Gilbert et al., 2013	C57BL/6 (inbred) mice	*G.v*. JCP8151B	NYC III	Anaerobic chamber	NR (16–18)	5 × 10^7^	Intravaginal (pipette)	39	36/39	Sialidase activity; epithelial exfoliation; clue-like cells, mucus degradation, uterine infection; absence of histologic inflammatory response
Hymes et al., 2013	C57BL/6 (inbred) mice	*G.v*. ARG37 (mouse-passaged ATCC14018)	NR	5% CO2	NR	5 × 10^6^	Intravaginal (5% gelatin)	10	10/10	DNase treatment reduced *G.v*. titers >10-fold
Sierra et al., 2018	CD-1(outbred) mice (pregnant)	*G.v*. ATCC14019	Tryptic Soy Broth + 5% Horse Serum	5% CO2	NR	2.5 × 10^7^^−^2.5 × 10^9^ (twice)	Intravaginal (pipette)	50-60	NR	*G.v*. detected in cervicovaginal fluid; increased cervical pro-inflammatory cytokines; increased IL-6 in amniotic fluid, cervical remodeling but no increase in preterm birth
Gilbert et al., 2019	C57BL/6 (inbred) mice	*G.v*. JCP8151B; *P. bivia* ATCC2903	NYC III (*G.v.)* CDC Anaerobe media + 10% laked sheep's blood (*P.b*.)	Anaerobic chamber	NR (16-18)	*G.v*. 8 × 10^7^ *P. bivia* 1-2 × 10^7^	Inravaginal (pipette)	31	23/31 (24hr)	Sialidase activity; epithelial exfoliation; increased vaginal *P. bivia* titers in presence of *G.v*.; increased uterine infection by *P. bivia* in presence of *G.v*; absence of inflammatory response
Gilbert et al., 2017	C57BL/6 (inbred) mice	*G.v*. JCP8151B; *E. coli* UTI89	NYC III (*G.v.)*	Anaerobic chamber	18	*G.v*. ~10^8^ (twice) *E. coli* 10^7^	Transurethral (catheter)	46	N/A	*G.v*. exposure triggered exfoliation of urothelial cells; emergence of *E. coli* reservoirs; *G.v*. kidney infection, kidney inflammation, more severe *E. coli* kidney infections
Trinh et al., 2011	ICR (outbred) mice	KCTC5096	BHI broth + yeast extract, maltose, glucose, 10% horse serum or general anaerobic medium	Sealed anaerobic jar	“Up to 36 hours”	1.2 × 10^5^	Intravaginal	6	NR	Increased vaginal TNF-α, IL-1β, and IL-6. Decreased IL-10. Increased iNOS, COX-2, and myeloperoxidase acitivity. Histological vaginal inflammation
Joo et al., 2011					48 h	1 × 10^6^				Increased vaginal TNF-α, IL-1β, IL-17a, COX-2, iNOS, and myeloperoxidase. Decreased IL-10. Histological vaginal inflammation
Jang et al., 2017										
Kim et al., 2019	C57BL/6 (inbred) mice	NR	General anaerobic medium	NR				7		Increased TNF-α and myeloperoxidase in vagina and uterus. Decreased IL-10 in uterus

### Vaginal Infection With *G. vaginalis* in Mice Results in Several Clinical Features of BV

**Figure 1:**
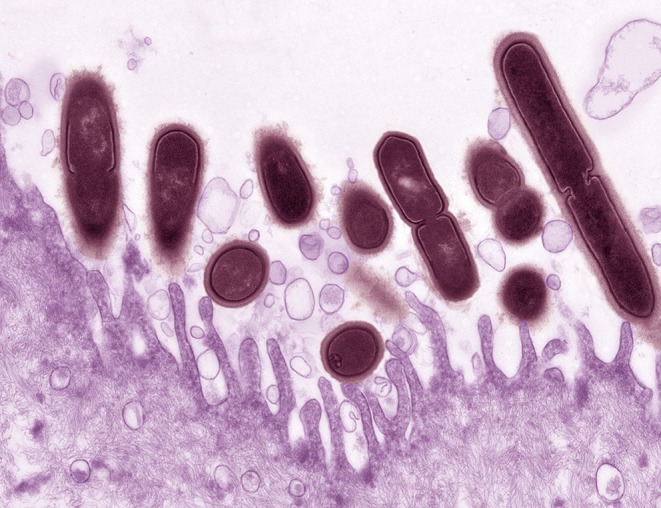
*Gardnerella vaginalis* (maroon) associated with the surface of a mouse vaginal epithelial cell (purple). Epithelial cells were collected from estrogenized mice by vaginal lavage with phosphate buffered saline. Epithelial cells were centrifuged, washed three times to remove endogenous bacteria then incubated for 4 h at 37°C *ex vivo* with *G. vaginalis* strain JCP8151B. Uranyl acetate staining was followed by transmission electron microscopy. Photo credit: Wandy Beatty. This image illustrates how the use of a small animal model can provide new resolution to aspects of BV that we appreciate, but do not fully understand. The pictured interaction provides evidence that in mice, as in women, *G. vaginalis* has an affinity with the vaginal epithelium. More broadly, it also supports the use of mouse models in reflecting at least some of the physiology we believe to occur in women.

In 2013, Gilbert et al. described a model of *G. vaginalis* vaginal inoculation into C57BL/6 mice (6-8 weeks of age) using a bacterial strain that had been recently isolated from a woman with BV (see Table 2) (Gilbert et al., 2013). The mice were treated with 17-β estradiol to bring them in proestrus, a technique that had previously been used to model vaginal infection with *Neisseria gonorrhoeae* (Jerse et al., 2011)*. G. vaginalis* colonized the mouse vagina and ascend into the uterine horns. *G. vaginalis* titers in vaginal washes and vaginal homogenates were strongly correlated, allowing for monitoring of *G. vaginalis* colonization in vaginal washes prior to the study's endpoint. Uterine and vaginal titers were also significantly correlated, suggesting that the degree of vaginal colonization was an important factor in ascending infection by the bacterium. In addition to the presence of *G. vaginalis* in vaginal fluid, several other BV-like features were detected in the model. There was a strong correlation in vaginal washes between the amount of live *G. vaginalis* and levels of sialidase activity, an enzyme that has been used as the basis for a point of care BV diagnostic test (Myziuk et al., 2003; Bradshaw et al., 2005). Mice inoculated with *G. vaginalis* also exhibited clue-like cells in vaginal washes, which were confirmed to be coated with *G. vaginalis* that were fluorescently labeled prior to inoculation. Although the literature often refers to epithelial exfoliation as an explanation for the presence of clue cells, this manuscript provided the first measurements of exfoliated epithelial cells, both in the mouse model and comparing women with and without BV. Significantly higher levels of exfoliated cells were observed in animals infected with *G. vaginalis* compared to mock-infected controls. Interaction between *G. vaginalis* and mouse epithelial cells was visualized by fluorescence microscopy (Figure 1). Likewise, women with Nugent-defined BV had significantly higher levels of exfoliated cells in Gram-stained slides compared to women without BV. Finally, as in women, the *G. vaginalis* vaginal colonization did not seem to induce classical signs of inflammation, as evidenced by the lack of neutrophil infiltration or edema (Gilbert et al., 2013). However, the lack of inflammation could also be a result of estradiol administration.

## *Gardnerella vaginalis* in Ascending Infection

*G. vaginalis* and other BV-associated bacteria are commonly isolated from human endometrial, placental, intraamniotic and perinatal infections (Berardi-Grassias et al., 1988; Hillier et al., 1988; Silver et al., 1989; Watts et al., 1990; Gibbs, 1993; Goldenberg et al., 1996; DiGiulio et al., 2010; DiGiulio, 2012; Petrina et al., 2019). In this section, we examine the ability of *G. vaginalis* to cause intrauterine infections after vaginal inoculation or elicit health complications associated with ascending infection during experimental infection.

### Intrauterine Inoculation in Pregnant Rabbits

Two studies have used a rabbit intrauterine infection model to investigate the impact of *G. vaginalis* in the upper reproductive tract during pregnancy (see Table 2). In this model, *G. vaginalis* (strain ATCC14018) was transcervically administered directly into each uterine horn by threading a cannula through the cervix (Field et al., 1993). The stated goal of these experiments was to determine if intrauterine infection by *G. vaginalis* would lead to preterm birth, fetal abnormalities, or maternal morbidity. The results indicated a significantly lower live-birth rate in the *G. vaginalis* inoculated rabbits compared to the saline-inoculated controls; however, there was not a greater incidence of preterm birth among animals in the infected group. In 88% of the infected animals, *G. vaginalis* could be detected in the amniotic fluid. Additionally, the fetal and placental weights on the viable fetuses were lower in the *G. vaginalis* infected group. The saline-inoculated uterine horns did not appear inflamed, but clinical signs of inflammation and histological deciduitis were observed in the uterine horns that received *G. vaginalis*. There was also significantly more evidence of neuropathology, including severe brain injury, in the *G. vaginalis* exposed fetuses. From these results, the authors conclude that *G. vaginalis* in the upper reproductive tract has pathophysiological consequences for both maternal and fetal tissues (Field et al., 1993).

Nearly a decade later, another study used the same transvaginal cervical cannulation model of *G. vaginalis* infection in pregnant rabbits (McDuffie et al., 2002). In this study, the animals were sacrificed prior to parturition on days 4, 5, or 6 post-inoculation, and a wide array of fetal and maternal tissues were cultured to detect live *G. vaginalis. G. vaginalis* was detectable (though titers were not reported) in the fetal brain, heart, and lung of some infected animals, as well as in maternal blood, uterine tissue, and amniotic fluid (see Table 2). Histological evidence of fetal brain damage was greater in the infected group, although the authors did not state whether the fetuses showing signs of neuropathology were also those that had detectable *G. vaginalis* titers in the brain. Additionally, the authors do not report the strain of *G. vaginalis* used in this study, or from where it was isolated (McDuffie et al., 2002). Of note, *G. vaginalis* has been reported as a cause of bacteremia in pregnant women and in at least one case, in a preterm neonate (Monif and Baer, 1976; Venkataramani and Rathbun, 1976; Reimer and Reller, 1984; Boggess et al., 1996; Agostini et al., 2003; Chen et al., 2018).

An important limitation of these two studies (Field et al., 1993; McDuffie et al., 2002) was that they bypassed the normal cervical barriers to ascending infection. As with many of the experimental studies of *G. vaginalis* infection, this study used a relatively high inoculum (see Tables 1, 2); it is unclear what amount of *G. vaginalis* exposure to the upper reproductive tract is physiologically relevant. It would have been informative if one of these studies had included an experimental control group in which “beneficial” vaginal bacteria (e.g., *Lactobacillus crispatus*) were introduced in the same manner (transvaginal cervical cannulation) to evaluate whether a non-pathogenic organism would be culturable from tissues or stimulate maternal and fetal immune responses when introduced in this manner. It is notable that other BV bacteria (e.g., *Prevotella bivia*) have been delivered using the same model, resulting in maternal fever and preterm delivery in up to 33% of animals (Gibbs et al., 2004). Intrauterine, intraperitoneal, or intravenous exposures to bacterial products such as lipopolysaccharide (Gram negative bacteria) (Fidel et al., 1998) or lipoteichoic acid (Gram positive bacteria) (Kajikawa et al., 1998) have also been used as models of preterm birth in pregnant mice.

### Vaginal Inoculation of *Gardnerella vaginalis* in Pregnant Mice Induces Cervical Remodeling

BV has been linked to increased risk of preterm birth, although the mechanisms linking such associations are still unclear (Svare et al., 2006). Sierra et al. (2018) used a pregnant mouse model of *G. vaginalis* infection to investigate the connection between BV and preterm birth. Timed-pregnant CD-1 mice were vaginally inoculated twice (on embryonic (E) days 12 and 13) with either 2.5 × 10^7^ or 2.5 × 10^9^ CFU of *G. vaginalis*. Animals were then sacrificed 48 h after the second dose (E15) or allowed to proceed to parturition. The authors did not observe a significant increase in preterm birth (defined as delivery any day before E18) in the *G. vaginalis* infected animals. There was also not a significant difference in pup weight or litter size between the control and infected animals. However, in those dams sacrificed on E15, the authors observed signs of inflammation and cervical remodeling following *G. vaginalis* inoculation. *G. vaginalis* was found in the lower reproductive tract at sacrifice, as determined by PCR. Although it is unclear what the detection limit was in these experiments, the authors were unable to detect the bacterium in the uterine horns, placenta, or fetal membranes. Histology revealed increased mucicarmine staining (interpreted as increased mucin, but other acidic glycans could also explain this result). The authors conclude that increased mucin production in pregnancy may reflect enhanced protection against ascending infection. We note that the ATCC14018 strain used in these experiments is negative for sialidase and does not encode the genes recently shown to be responsible for sialidase activity in cultured isolates (Robinson et al., 2019). Thus, it is also possible that this strain lacks the ability to cause ascending infection due to an inability to engage in mucin degradation. There was a significant increase in IL-6 protein levels in the cervicovaginal fluid of infected animals as compared to the controls. Interestingly, there was also increased IL-6 in the amniotic fluid (AF) of infected animals, despite the apparent lack of upper-reproductive tract colonization by *G. vaginalis*. Infected mice also had increased soluble E-cadherin in the cervicovaginal space, a biomarker of cervical epithelial remodeling, and increased expression of Tff-1 gene (Sierra et al., 2018), which is also thought to be important in the remodeling process (Akgul et al., 2014). There was also increased transcript levels of the pro-inflammatory cytokines IL-8, IL-1β, and IL-10 in the cervices of infected mice. The authors also observed dispersion of collagen fibers in the cervices of infected dams. Biomechanical testing showed that cervices of *G. vaginalis* infected animals displayed significantly lower modulus and higher maximum strain, but displayed no difference from the control group in tissue cross sectional area, maximum load, stiffness, or maximum stress. These data provide evidence in support of the conclusion that cervical softening may be “occurring faster/earlier” in response to *G. vaginalis* (Sierra et al., 2018). While remodeling of the cervical epithelium is a normal part of pregnancy as the body prepares for parturition (Timmons et al., 2010), early induction of this remodeling by *G. vaginalis* could contribute to preterm birth.

### *Gardnerella* Infection and Vaginal Inflammation

As previously discussed, there is some controversy surrounding BV's classification as an inflammatory or non-inflammatory condition. Most cases of BV lack clinical signs of overt inflammation such as swelling and redness (Mitchell and Marrazzo, 2014), which seems at odds with studies that report increased levels of inflammatory cytokines (Cauci et al., 2002, 2008; Hemalatha et al., 2012). To the best of our knowledge, cytokine responses to *G. vaginalis* infection have not been extensively studied using animal models. One exception is the study just discussed by Sierra et al. in which authors found elevated transcripts of several inflammatory cytokines in *G. vaginalis* infected mice (Sierra et al., 2018). Another notable exception is a group of studies published by a single group, which investigate probiotic strategies to treat BV using a mouse model of *G. vaginalis* infection (Joo et al., 2011; Trinh et al., 2011; Jang et al., 2017; Kim et al., 2019). These studies assessed immune markers of vaginal inflammation in response to inoculation with *G. vaginalis*, to determine if these markers were lower in animals receiving probiotics. These papers performed intravaginal inoculation with the *G. vaginalis* strain KCTC5096 to model BV in β-estradiol treated mice (Joo et al., 2011; Trinh et al., 2011; Jang et al., 2017) [note: bacterial strain not reported in] (Kim et al., 2019). The mouse strains used were ICR in two of the papers (Joo et al., 2011; Trinh et al., 2011), and C57Bl/6 in the others (Jang et al., 2017; Kim et al., 2019).

Overall, all four studies report finding increased levels of pro-inflammatory cytokines such as TNF-α, IL-1β, and IL-6 (determined by ELISA) in the vaginal tissue of infected mice as compared to non-infected controls (Joo et al., 2011; Trinh et al., 2011; Jang et al., 2017; Kim et al., 2019). The authors also reported that IL-10, often regarded as an anti-inflammatory cytokine (Couper et al., 2008), was consistently present at lower levels in *G. vaginalis* infected mice compared to controls. In addition to cytokines, the authors examined evidence of *G. vaginalis*-induced inflammation by assessing myeloperoxidase, iNOS, and COX-2 levels in the vaginal tissues of infected mice. The authors report that mice inoculated with *G. vaginalis* had higher levels of these inflammatory markers compared to uninfected controls. Additionally, when histology was performed on vaginal tissue, there appeared to be substantial edema and immune cell infiltrate into the superficial mucosal layers (Joo et al., 2011; Trinh et al., 2011).

A caveat to the findings of these studies is that some aspects of the methodology are obscure. For example, it is not always clear if the control group of uninfected mice were given β-estradiol treatment. If they were not, it becomes impossible to compare the infected vs. non-infected groups, as any differences in inflammatory markers or histopathology could simply be the result of only one group receiving hormone treatment. Additionally, in at least one of the studies, the inocula of *G. vaginalis* seems to be inconsistent between figures (Jang et al., 2017). None of the data reported in these four publications show individual data points to represent the mice in each group and the routine use of means and standard deviation might make these data more sensitive to skewing by outliers. Finally, several of the reported interpretations of increased vaginal inflammation rely on immunoblots that were not quantified and histological images that were not scored (Joo et al., 2011; Trinh et al., 2011; Jang et al., 2017).

The question of BV and inflammation remains a tricky one. Future *in vivo* studies aimed at untangling this mystery will need to be rigorous about having appropriate controls and reporting the data and methods in clear detail.

## Understanding How *G. vaginalis* MAY Influence the Growth or Pathogenesis of Other Bacteria

Despite Gardner and Dukes early assertions that *Gardnerella* was the sole causative agent of BV, this is not a universally accepted hypothesis (Hickey and Forney, 2014; Schwebke et al., 2014a,b). Another hypothesis is that multiple species of bacteria are needed to generate the features and complications that have been associated with the condition (Muzny et al., 2019a). There is also evidence, however, that *G. vaginalis* may still have a role in the development of features that it does not directly cause, and may do so by impacting the abundance or pathogenesis of other organisms.

### The Characteristic Fishy Odor of BV Cannot Be Solely Attributed to *G. vaginalis*

In 1979 Chen et al. identified seven amines in the vaginal fluid of women with BV (then NSV) that were absent in the fluid of healthy controls. The authors theorized that these amines likely contribute to the “fishy” odor associated with the condition, and showed that the amines could be produced *in vitro* by the vaginal bacteria isolated from NSV patients. However, while these mixed vaginal communities were able to produce amines, *G. vaginalis* isolates alone did not do so under the conditions studied, suggesting that one of the major diagnostic features of BV cannot be solely attributed to the presence of *Gardnerella* (Chen et al., 1979). This finding is supported by a more recent report from Nelson et al. (2015), which sought to identify the vaginal bacteria capable of producing the BV-associated amines. The authors looked for homologs of characterized biogenic amine-synthesizing proteins in the genomes of common vaginal bacterial taxa. They found no evidence of any predicted genes encoding biogenic amine-synthesizing proteins in the four *G. vaginalis* strains they investigated (Nelson et al., 2015).

If *G. vaginalis* is unable to produce the amine species characteristic of BV, then it is necessary to re-examine interpretations from some of the first *Gardnerella* infection models. The early human experiments by Criswell et al. (1969) found that vaginal inoculation of pure *G. vaginalis* cultures could replicate the clinical symptoms of “*H. vaginalis* vaginitis” as described by Gardner and Dukes. These symptoms included the “characteristic offensive odor” of the vaginal discharge (Criswell et al., 1969). Assuming that the authors are describing the same “fishy” odor as the Amsel criteria, this means that inoculation with *G. vaginalis* was able to lead to the presence of various amines in vaginal fluid. If *G. vaginalis* itself is unable to produce these amines, this would suggest that colonization of the vaginal tract by *G. vaginalis* also encourages the growth of amine-producing bacterial species. Even at the time, Chen et al. (1979) noted a possible symbiosis between *G. vaginalis* and amine-producing bacteria, observing that while *G. vaginalis* cannot produce amines *in vitro*, it does release large concentrations of pyruvic acid and amino acids during growth. Conversely, amino acids and pyruvic acid were depleted in the media of the NSV mixed vaginal communities that were able to produce amines, indicating the possibility that metabolites produced by *G. vaginalis* can be consumed by amine-producing vaginal bacteria (Chen et al., 1979).

Currently the BV field has identified several vaginal bacterial taxa that have either been shown to produce amines *in vitro* or have predicted amine-synthesizing proteins, including *Prevotella, Dialister*, and *Eggerthella* species (Pybus and Onderdonk, 1997; Nelson et al., 2015). Srinivasan et al. (2012) showed that the presence of these taxa in the vaginal microbiome was associated with the Whiff test, lending creed to their potential role as amine producers. Notably, Srinivasan et al. (2012) also found that the Whiff test was associated with the presence of *G. vaginalis*. This finding seems to provide additional evidence to the possible symbiosis between *G. vaginalis* and amine-producers in the vaginal environment (Srinivasan et al., 2012).

### *G. vaginalis* Encourages Ascending Uterine Infection by *Prevotella bivia* in Non-pregnant Mice

In 2019, Gilbert et al. described an estradiol-treated mouse co-infection model with *G. vaginalis* and *Prevotella bivia* (Gilbert et al., 2019). *Prevotella* species are prevalent in the vaginal microbiome of women with BV (Srinivasan et al., 2012). Additionally, like a number of other BV-associated organisms, vaginal colonization by *P. bivia* has been linked to higher rates of preterm birth (Krohn et al., 1991). In the reported model, both *G. vaginalis* and *P. bivia* were able to colonize mouse vaginas on their own, but co-inoculation with *G. vaginalis* led to higher *P. bivia* vaginal titers 1-day post-infection, compared to animals that were inoculated with *P. bivia* alone. Consistent with the idea of reciprocal interactions between *Gardnerella* and *Prevotella* (as discussed in section Interaction Between Gardnerella and Curved Rods Generates Features of BV in Grivet Monkeys above), the titers of the two organisms during vaginal colonization were highly correlated (Spearman r = 0.9636). Additionally, *P. bivia* titers in the uterine horns of co-infected animals were ~20 times higher than in mono-infected animals at 2 days post-infection. This finding suggests that *G. vaginalis* enhances the ability of *P. bivia* to cause ascending infection of the reproductive tract. In this model, *G. vaginalis* titers in the vagina and uterine horns were not significantly different between mono and co-infected groups. Consistent with the *G. vaginalis* inoculation model reported in 2013, no clinical signs of inflammation were observed in the vaginas or uterine horns of mono or co-infected animals (Gilbert et al., 2013, 2019).

### Interaction Between *Gardnerella* and Curved Rods Generates Features of BV in Grivet Monkeys

In the 1980s it was reported that women with BV often had motile, anaerobic curved rods (CR) in their vaginas. In 1984, Mardh et al. vaginally inoculated grivet monkeys with both *G. vaginalis* and CR isolates to determine if the clinical features of BV could be replicated in a co-infection primate model (see Table 1 for comparison to other human and non-human primate experiments). This group used a short morphotype (SCR) and long morphotype (LCR) (Mårdh and Møller, 1984). While we cannot say for certain, it seems highly likely that these CR morphotypes were in fact distinct species of *Mobiluncus* that are commonly present in women with BV. Two morphotypes of *Mobiluncus* can be distinguished by Gram-stain examination: small (1.7 um) Gram-variable cells that correspond to *Mobiluncus curtisii* and large (2.9 um) Gram-negative cells that correspond to *Mobiluncus mulieris* (Dworkin et al., 2006). Another possibility is that CR morphotypes on Gram stained slides may be BVAB1 (a member of the family *Lachnospiracea* and order *Clostridiales*) rather than *Mobiluncus* (Srinivasan et al., 2013). However, given that modern microbiologists have not yet succeeded in cultivating BVAB1, it seems more likely that what was isolated and used in the Grivet monkey model was in fact *Mobiluncus*.

In the Grivet model, *G. vaginalis* alone was unable to colonize, as the authors could not recover it from the mono-infected animals at 6 days post-infection. Conversely, both morphotypes of CR could be isolated back out of mono or co-infected animals for the entire 37-days observation period. One monkey that had been inoculated with LCR was still culture positive after 9 months. However, no mono-infection with any of the three organisms (SCR, LCR, or *G. vaginalis*) caused the animals to develop any clinical signs of BV (clue cells, increased vaginal discharge, or elevated vaginal pH). Co-infection with SCR and *G. vaginalis* also did not yield any clinical signs of BV, and while SCR could be recovered during the entire 37 days, *G. vaginalis* was never re-isolated. However, animals that were co-infected with *G. vaginalis* and LCR developed “profuse vaginal discharge” that began 5 days after infection, which lasted through the entire 37-days observation period. LCR could be isolated back out of the co-infected animals during the entire 37 days, and *G. vaginalis* was recoverable until day 12. Co-infection with *G. vaginalis* and LCR was the only condition in which *G. vaginalis* was ever recoverable, and the only condition in which the profuse discharge was observed. No clue cells or vaginal inflammation were observed under any of the conditions (Mårdh and Møller, 1984).

### *G. vaginalis* Triggers Kidney Injury and Recurrent Urinary Tract Infection by *Escherichia coli*

In addition to being present in the vagina, multiple studies have isolated *G. vaginalis* from urine, including those that used methods to collect urine that limit contamination by peri-urethral and vaginal bacteria (e.g., suprapubic needle aspiration or transurethral catheterization) (Pearce et al., 2014, 2015; Malki et al., 2016; Thomas-White et al., 2016; Gottschick et al., 2017; Kramer et al., 2018). Although most routine methods for urine culture will not detect *Gardnerella*, studies that have used appropriate isolation conditions suggest that the bladder is either transiently exposed to *G. vaginalis* or that the organism may colonize the bladder in some women [see Kline and Lewis (2016) and references therein]. In one study, *G. vaginalis* was cultured from (needle) aspirated urines from 20/33 (60%) patients with chronic atrophic pyelonephritis (a.k.a. reflux neuropathy) compared to only 2/35 healthy controls (9%) (Fairley and Birch, 1983). Of 61 aspirates from men that were tested, none yielded growth of *G. vaginalis*, while about 1/3 of women tested had *G. vaginalis* in aspirated urine. Interestingly, women with culture-positive (*G. vaginalis, U. urealyticum*, or other species) aspirated urine also had squamous epithelial cells covered in bacteria, bearing a close resemblance to clue cells seen in BV. Another study of hospital inpatients reported that individuals from whom *Gardnerella* was isolated from urine were more likely to have a history of recurrent UTI and current pyelonephritis (Josephson et al., 1988a). Together, these clinical data support further investigations of the effects of *Gardnerella* in the urinary tract.

Several reports suggest that women with BV are more likely to experience UTI (Harmanli et al., 2000; Hillebrand et al., 2002; Sharami et al., 2007; Sumati and Saritha, 2009; Amatya et al., 2013; Gautam et al., 2015). One recent study reported a mouse model to test the hypothesis that *G. vaginalis* interactions with the bladder may contribute directly to the association between BV and UTI (Gilbert et al., 2017). Mice first received transurethral inoculation of *Escherichia coli*, the most common cause of bladder and kidney infections. This resulted in the formation of intracellular reservoirs that persist following clearance of the bacteria from urine, as had been shown previously (Mysorekar and Hultgren, 2006). Mice were then inoculated twice with *G. vaginalis. G. vaginalis* did not establish lasting colonization in the bladder, and nearly all mice had undetectable titers by 12 h following exposure. However, scanning electron microscopy and histological analysis of the bladders, as well as urine cytology, revealed that even a transient exposure to *G. vaginalis* induced urothelial exfoliation. In the subsequent days following *G. vaginalis* exposure, *E. coli* re-emerged from latent reservoirs back into the urine to cause recurrent UTI that was accompanied by a neutrophilic response. This phenotype was not observed when the mice were transurethrally inoculated using identical methods in parallel, with similar numbers of *L. crispatus*. This condition was used to model bladder exposure to vaginal bacteria from a woman without BV. Additionally, bladder exposure to *G. vaginalis* increased the susceptibility of the mice to developing severe (kidney and systemic) *E. coli* infections. This is consistent with a clinical study which showed that kidney infection was more common among female inpatients who had detectable *G. vaginalis* in their urine (Josephson et al., 1988b). Gilbert et al. (2017) also demonstrated that transurethral exposure to *G. vaginalis* was sufficient to cause acute kidney injury that was a direct result of inflammatory signaling via the IL-1 receptor and occurred even in the absence of *E. coli* (Gilbert et al., 2017). These findings, in conjunction with clinical data, suggest that further investigation into the ability of *G. vaginalis* to induce urologic pathology is necessary. As with the other models discussed above, it will be interesting in future studies to learn if different species, subspecies, or strains of *Gardnerella* (see more below) are able to generate the different observed pathophysiologic features in the urinary tract.

## Discussion

### Taxonomic Diversity and Pathogenesis of *Gardnerella*

Since the initial discovery of *G. vaginalis* (then *H. vaginalis*) (Gardner and Dukes, 1954), many efforts have been made to taxonomically sub-divide and identify isolates with the greatest virulence potential (Piot et al., 1980, 1984; Pleckaityte et al., 2012). Recent comparative genomic studies indicate that *G. vaginalis* comprises at least four distinct phylogenetic clades/subtypes (Ahmed et al., 2012; Malki et al., 2016). Recently, one manuscript proposed a few new species names within the genus *Gardnerella* (Vaneechoutte et al., 2019). However, the broader clade designations contain multiple proposed *Gardnerella* species, although names have only been proposed for some. Regardless of the exact taxonomic scheme used, the genomic diversity among *Gardnerella* isolates is substantial. Early reports have suggested that the core genome shared by all *G. vaginalis* isolates consists of only 25% of the total genes in the *G. vaginalis* pangenome (Ahmed et al., 2012). The fact that there are so many accessory genes among *G. vaginalis* clades supports the *hypothesis* that the clades may occupy distinct niches characterized by unique affiliations with other microbes, communities, or host factors. Although different studies have not always given consistent results, several clinical studies implicate clade 1 and/or clade 2 strains in BV status, recurrence, and other adverse outcomes linked with BV, such as preterm birth. Conversely, clades 3 and 4 lack certain predicted virulence factors and may be less pathogenic (Balashov et al., 2014; Callahan et al., 2017; Hilbert et al., 2017; Goltsman et al., 2018; Janulaitiene et al., 2018; Plummer et al., 2019). Interestingly, specific clades have also been linked with sexual practices. For example, one recent study found that clade 1 was associated with higher numbers of recent sexual partners while clade 2 was associated with penile-vaginal intercourse and sharing of sex toys with female sex partners (Plummer et al., 2019).

The earliest suggestions that women with BV may harbor multiple subtypes of *G. vaginalis* came in 1990 when Briselden and Hillier tested vaginal specimens for different “biotypes” of *G. vaginalis* (Briselden and Hillier, 1990). Following the more recent availability of genome sequences and clade definitions, several literature reports have confirmed that multiple *G. vaginalis* clades often co-exist in the vagina in women with BV (Balashov et al., 2014; Janulaitiene et al., 2018; Plummer et al., 2019; Shipitsyna et al., 2019). Because of this complexity, it is difficult based on observations of clinical samples alone to make definitive assertions about which clades may be involved in different phenotypes or pathologies associated with BV.

Experimental models in animals (in the spirit of Koch's postulates) are needed to begin unraveling how each clade may interact with the host, other organisms, and/or each other to elicit features and health complications associated with BV. Clade identifications are only available for some of the strains used in previous experimental studies conducted to date. The most common strains used in animal models have been ATCC14018 (clade 1) and JCP8151B (clade 2, now designated *G. piotii*), both of which were sufficient to cause features and/or complications linked with BV in human and animal models (see Tables 1, 2). Some of the limitations of these models and their conclusions are discussed in the paragraphs that follow.

### Limitations of Animal Models

Mouse models of *G. vaginalis* infection have become much more prevalent over the last decade and have shed light on a number of interesting phenomena concerning *G. vaginalis* and BV. However, there are clear limitations with these models. As with the primate models, laboratory mice do not have a *Lactobacillus* dominant microbiome. Therefore, it is not surprising that the increase in vaginal pH associated with BV has not been achieved in these models (Miller et al., 2016). Additionally, there are likely to be differences in the endogenous vaginal microbiomes depending on the strain of mouse and the facility where they are raised. Differences in the mouse strain, housing conditions, and/or vaginal microbiome could strongly impact susceptibility and response to *G. vaginalis* infection. For example, Teixeira et al. (2012) observed “inflammatory lesions” upon *G. vaginalis* infection in their germ-free mice, while Gilbert et al. (2013, 2019) have specifically noted a lack of inflammatory response in their model using C57Bl/6 mice raised under conventional conditions (Teixeira et al., 2012; Gilbert et al., 2013, 2019). Additionally, many of the mouse models require β-estradiol administration to bring mice into pro-estrus for the duration of the experiment and avoid the PMN-rich post-ovulation period of the estrus cycle (Gilbert et al., 2013). A potential limitation is that normal immune responses to *G. vaginalis* infection may be altered by the use of β-estradiol. Another potential limitation of mouse models is that the vaginal epithelium is more highly keratinized in mice (during pro-estrus) than in humans, which may influence the normal course of *G. vaginalis* infection (Sierra et al., 2018). Finally, previous work had shown that the *G. vaginalis* toxin vaginolysin (a cholesterol-dependent cytolysin) is species-specific and requires the presence of human CD59 (Gelber et al., 2008). It is not clear whether this finding applies to other primate models, but nevertheless, the development of a humanized mouse expressing human CD59 might improve the *G. vaginalis* mouse infection model. Perhaps the biggest limitation of experimental studies in humans or animals is that they rarely consider that the presence of the endogenous vaginal microbiome is likely to discourage colonization by exogenous organisms that are introduced, a phenomenon known as colonization resistance. The use of non-human primates in research presents additional challenges, expense, and ethical considerations, but may be worthwhile to better understand the microbial ecology and interactions with the host mucosa in BV. It has been noted that the vaginal microbiomes of many of the species of non-human primates for whom such studies have been performed have communities that resemble BV (Uchihashi et al., 2015; Zhu et al., 2015; Obiero et al., 2016; Miller et al., 2017). Thus, while there are greater obstacles to their use, nonhuman primates may also offer important advantages over other model systems.

### Ethics of Human Experimentation

When the early human infections with *G. vaginalis* were performed, the experimenters had little understanding of the pathology of BV. The risks and adverse health outcomes associated with BV were unknown at the time, and the condition was thought to be relatively benign. Indeed, in their hallmark 1955 paper, Gardner and Dukes express their belief that BV is “physically and esthetically objectionable,” but that “the disease is not a serious one.” Additionally, they wrote that “therapeutically, [BV] constitute[s] no difficult problem” (Gardner and Dukes, 1955) which is in clear contrast to more recent literature on the difficulty treating BV and the high rates recurrence (Bradshaw and Brotman, 2015). This erroneous belief in the banality of BV explains how groups in the 1950s and 60s were comfortable performing vaginal inoculations of *G. vaginalis* into women (Gardner and Dukes, 1955; Criswell et al., 1969). In light of our current understanding, it can be clearly argued that these experiments represented an unacceptable risk for the subjects that in today's scientific environment would need to be clearly articulated during informed consent and could result in outright rejection by institutional review boards. While these early papers provide useful insight into *G. vaginalis* and BV, their ethical dubiousness also act as a cautionary tale in the dangers of misguided assumptions in the pursuit of further understanding.

This lesson is an important one to remember, especially considering that human experimentation in BV research still occurs today. Now however, the experiments are aimed at directly treating BV and improving quality of life for those women afflicted. In 2019 Lev-Sagie et al. reported the first vaginal microbiome transplantation (VMT) performed in human women. The transplants were from three BV-negative donors with *Lactobacillus crispatus*-dominated vaginal microbiomes. The five recipients were women with intractable BV who experienced recurrent symptomatic episodes, despite antibiotic treatment, and so were good candidates for alternative therapy. The recipients all received antibiotic treatment for 5–7 days immediately prior to the first VMT. Vaginal discharge from the donors was collected and introduced into the recipients, and the recipients received clinical follow-up to assess BV symptoms for 5–21 months following VMT. Four of the five recipients experienced full long-term remission (defined as absence of symptoms, an Amsel score of 0, and the presence of a *Lactobacillus* dominated microbiome), although three of the four required multiple rounds of VMT. The fifth woman experienced partial symptom improvement, although her vaginal microbiome was not lactobacilli-dominated at the end of the observation period (Lev-Sagie et al., 2019).

These results overall appear promising, and unlike in the human experiments of the 1950s and 60s, the authors of this study carefully screened donors for STIs and other important infections such as hepatitis and cytomegalovirus. However, the authors themselves also acknowledge that there is always the possibility of unforeseen risk with VMT—after all, we still know relatively little about the vast majority of the microbes that live in this niche. For this reason, Lev-Sagie et al. emphasize that VMT should be considered only as a last resort for intractable BV where quality of life is severely disrupted and multiple treatment options have failed (Lev-Sagie et al., 2019).

Finally, we note the need for consistency in diagnostic terms for BV that are used by academia and industry as well as the need to provide specific methods used to define such diagnoses.

### Have Koch's Postulates Been Satisfied That *G. vaginalis* Is a Cause of BV?

Koch's original postulates established a microorganism is the cause of a disease if it was (1) found in all diseased, but not healthy individuals, (2) could be isolated and grown in pure culture, (3) caused disease when introduced into an experimental host, and (4) could be re-isolated and re-identified from the infected experimental host. Of course, arguments have been advanced by Koch himself, and many others since, in favor of revising the original postulates for biological and technical reasons as it became clear that they were too rigid in their original form (see below).

#### Is *Gardnerella* Found in All Cases of NSV/BV but Not Among Healthy Women?

Over the years, scientists have used different methods to detect and identify *Gardnerella* and different criteria to define BV, leading to somewhat different conclusions. In the first published studies, Gardner and Dukes found that *G. vaginalis* was detected in >90% of women with clinical signs we now recognize as BV, but never in apparently healthy control women (Gardner and Dukes, 1955). By the 1980's, the use of human blood bilayer media and other culture techniques had improved the ability to culture “*Gardnerella*” and led to a higher proportion of “normal” women appearing positive for *Gardnerella* (Totten et al., 1982; Hillier, 1993). However, culture-based studies in the 1980s and more recent quantitative PCR methods seem to agree that *Gardnerella* is found at orders of magnitude higher levels in women with BV compared to those without the condition (Sheiness et al., 1992; Balashov et al., 2014). This first of Koch's postulates has been modified for causative agents of some bacterial infections in light of findings that they can be harbored by a subset of individuals without causing symptoms or harm (e.g., *Haemophilus influenza, Staphylococcus aureus, Streptococcus pneumoniae, Streptococcus agalactiae*, etc.) (Fredricks and Relman, 1996; Duell et al., 2016; Krismer et al., 2017; Shabayek and Spellerberg, 2018; Weiser et al., 2018). In fact, Koch himself realized this upon the discovery that some individuals carried the causative agents of cholera and typhoid without apparent signs of disease (Evans, 1976). Thus, it is also possible that carrier states may exist for *Gardnerella*. Two alternate explanations for the presence of *Gardnerella* in women without BV could be that the strains in question may have fewer virulence factors (see the section above on *Gardnerella* taxonomy) or are kept “in check” by strains of beneficial bacteria with particularly powerful antimicrobial activities.

#### Can *Gardnerella* Be Isolated in Pure Culture and Re-Isolated From Infected Hosts?

Since its original description in the 1950s, the isolation and growth of *Gardnerella* in culture has been reported using several methodologies, including different culture media (see Tables 1, 2) and use of different atmospheric conditions (anaerobic, 5% CO_2_). Broadly speaking, it is not known whether the reported culture conditions for preparing *G. vaginalis* inocula used in experimental studies result in expression of the factors needed for colonization/infection. Experimental studies in humans (Criswell et al., 1969), primates (Mårdh and Møller, 1984), and mice (Gilbert et al., 2013) showed that when signs of BV were present, *G. vaginalis* was recoverable. One exception to this was the finding in pig-tailed macaques that *G. vaginalis* was recoverable from 10/10 inoculated individuals but did not result in the appearance of clue cells, more basic pH, or change in non-volatile acids (Johnson et al., 1984). As discussed elsewhere, based on the absence of dominant lactobacilli in these animals, it is arguable that the latter two signs may not be possible to generate in this system.

#### Can *Gardnerella* Reproduce Features of BV in Experimental Models?

Only two studies in the literature inoculated *Gardnerella* into human volunteers. Although the first experiments from Gardner and Dukes (1955) claimed “proof of pathogenicity,” these studies provide little evidence for this conclusion (see Section Vaginal Inoculation in Women) (Gardner and Dukes, 1955). It is hard to know what the authors did in these experiments since they report no detailed culture conditions or bacterial strain names. There was not a control group of women left uninoculated to determine if women might spontaneously develop signs of BV or become colonized with *Gardnerella*.

**Figure 2:**
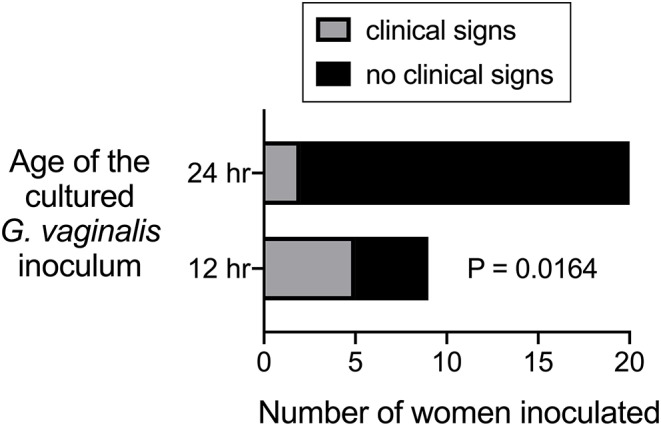
Data from Criswell et al. (1969) plotted and analyzed to evaluate statistical difference between ability of 12 h vs. 24 h *G. vaginalis* cultures to cause signs of BV when inoculated into women.

The second of the two experimental studies performed in humans was also performed by Gardner and Dukes, but involved another co-author (Criswell). One of the interesting questions evaluated by Criswell et al. (1969) was whether *Gardnerella* strains grown in culture for 12 or 24 h resulted in clinical signs of BV when “poured” into the vaginas of 29 women. About half of these women were inoculated with the strain ATCC14018, which caused clinical signs of BV in most of the women when the inoculum was prepared from a 12-h culture. In contrast, the same strain grown for 24-h did not cause signs of BV in any of the inoculated individuals. Three other strains were used in this paper to inoculate another 15 women. Although clade designation of these strains is not known, 24-h cultures were used and only two of fifteen women were found to have signs of BV (Criswell et al., 1969). Although statistical analysis was not presented by the authors, our analysis of results from the 29 women inoculated shows that those receiving 12 h cultures were significantly more likely to develop signs of BV compared to those who received 24 h cultures (Figure 2). This raises the question of whether 24 h cultures of *Gardnerella* under the conditions used were viable for colonization.

Later studies that investigated signs of BV in primates inoculated with *Gardnerella* did not provide substantial evidence in support of *Gardnerella* as a causal factor in the development of BV, but had several important limitations. First, none of the strains used have survived to the present day so we cannot use molecular techniques to confirm their identity as *G. vaginalis* or determine to what clade they belong. Additionally, it is not always clear how the bacteria were cultured for preparation of the inoculum (medium or atmospheric conditions used were not fully reported). The Johnson et al. (1984) and Mårdh and Møller (1984) studies report using older cultures of *Gardnerella* (24 or 48 hour cultures) (Johnson et al., 1984; Mårdh and Møller, 1984). As stated above, although “increased pH” was one of the main foci of the investigations, the primates used in these studies typically already have more neutral vaginal pH at baseline (before inoculation). Thus, it may not be possible to make the vaginas of these animals more basic. Later studies have illustrated that non-human primates harbor microbiomes that contain diverse taxa and typically are not characterized by dominant lactobacilli (Stumpf et al., 2013). With that said, it is not always clear how interactions with humans may change the landscape of wild microbiomes, as recent studies in mice have revealed (Rosshart et al., 2017). It is noteworthy that 10/10 pig-tailed macaques developed colonization with *Gardnerella* in the Johnson et al. (1984) study, but did not develop signs of BV (Johnson et al., 1984). But, as we do not know what taxonomic lineage the strains used in this study belong to, the lack of BV signs could also reflect a less virulent isolate was used.

We argue that the most definitive conclusions regarding whether *G. vaginalis* experimental administration reproduces features and complications associated with BV in women can be derived from experiments performed using three strains: ATCC14018, ATCC14019, and JCP8151B. Experimental studies performed using the clade 1 strain ATCC14018 showed that this strain yielded clinical signs of BV in 5/9 women when 12-h cultures were used for inoculation (Criswell et al., 1969) (see Figure 2). More than four decades later, one study may have provided an explanation by showing that extracellular DNA produced by *G. vaginalis* contributes importantly to the production of biofilms and is only present in the cell-free supernatant of early cultures (Hymes et al., 2013). Another *G. vaginalis* clade 1 strain, ATCC14019 was originally isolated by Gardner and Dukes and is nearly identical in genome sequence to ATCC14018. Administration of ATCC14019 vaginally into pregnant mice resulted in evidence of cervical remodeling and increased IL-6 in the vagina and amniotic fluid (Sierra et al., 2018), consistent with findings of elevated IL-6 in women with BV (Yudin et al., 2003; Campos et al., 2012) and associations of clade1/2 *G. vaginalis* with preterm birth (Callahan et al., 2017). In another mouse model, the clade 2 strain JCP8151B was shown to result in colonization and live recovery of *Gardnerella*, clue-like cells with adherent *Gardnerella*, an epithelial exfoliation response similar to that seen in humans, and higher levels of the hydrolytic enzyme sialidase together with biochemical evidence of mucus degradation, similar to that seen in BV (Gilbert et al., 2013; Lewis et al., 2013). The recent suggestions for new species names for subsets of *Gardnerella* strains seems to place the JCP8151B strain within the subset of clade 2 strains being referred to as *Gardnerella piotii* (Hill et al., 2019; Vaneechoutte et al., 2019). In short, the experimental evidence points to the conclusion that *G. vaginalis* and *G. piotii* (if this naming scheme is adopted by the field) can cause clinical signs of BV when introduced into human and animal hosts.

#### Can *Gardnerella* Influence the Pathogenesis of Other Organisms in Experimental Models?

Women with BV appear to be more susceptible to a wide variety of other infections, including infections of the lower reproductive tract (Brotman et al., 2010), the upper reproductive tract (Ádám et al., 2018), and the nearby urinary tract (Harmanli et al., 2000). However, we still know relatively little about which organisms in BV might confer these increased risks or the mechanisms leading to disease. In addition to findings that certain strains of *Gardnerella* can reproduce features of BV in experimental models, there is also evidence to support the conclusion that *Gardnerella* can influence whether other organisms cause pathophysiology. Specifically, vaginal colonization by the JCP8151B strain caused mice to experience higher titer ascending uterine infections by *Prevotella bivia* compared to animals that received only *P. bivia* (Gilbert et al., 2019). The JCP8151B strain was also able to trigger recurrent UTI caused by uropathogenic *E. coli* that re-emerged from long lasting reservoirs inside epithelial cells in response to bladder exposure to *Gardnerella*, but not *Lactobacillus crispatus* (Gilbert et al., 2017).

In conclusion, we have only scratched the surface in understanding how microbes coordinate the biological processes that initiate and perpetuate BV. Nevertheless, experimental models have given us key insights into the causal relationship of *Gardnerella* with BV. Together, the studies reviewed here suggest that *Gardnerella vaginalis* can, under some conditions, recreate some of the features and complications that have been associated with BV. Although we do not yet understand the precise molecular mechanisms, additional experimental studies suggest that *Gardnerella vaginalis* may change the host landscape in a way that makes other organisms more likely to colonize or cause disease. Future studies should expand and optimize small animal models to further refine our understanding of how species, subspecies, and strains of BV bacteria act alone and in concert with other microbes to overcome the healthy microflora and lead to poor health outcomes.

## Author Contributions

AL paid the page charges from grant funds. All authors participated in writing this manuscript and approved of the final version.

## Conflict of Interest

The authors declare that the research was conducted in the absence of any commercial or financial relationships that could be construed as a potential conflict of interest.
